# Field experimental study on ground treatment of high fill embankments over expansive soils

**DOI:** 10.1038/s41598-025-14911-2

**Published:** 2025-08-20

**Authors:** Bin Hou, Faning Dang, Weihua Ding, Haibin Xue, Hong Zhan

**Affiliations:** 1 Xi’an University of Technology, Xi’an, 710048 Shaanxi China; 2 SCEGC Mechanized Construction Group Co., Ltd., Xi’an, 710032 Shaanxi China

**Keywords:** High fill embankment, Expansive soil, Dynamic compaction, Sand compaction pile, In-situ experimental study, Engineering, Civil engineering

## Abstract

In valley regions where high-fill embankments are constructed, inadequate foundation bearing capacity is a frequently encountered challenge. At the Ankang Airport relocation site, expansive soils primarily originating from the Upper to Middle Pleistocene are widely distributed, which introduces substantial safety concerns for the stability of high-fill embankments. Prior to large-scale filling, a field experimental site was selected within the project area to conduct in-situ tests for ground improvement. Two ground reinforcement techniques—dynamic compaction and gravel pile compaction using driven casing—were implemented and assessed. Evaluations of physical and mechanical properties, including plate load and standard penetration tests (SPT), were carried out before and after the improvement procedures. Test results showed that the natural foundation soil had a characteristic bearing capacity of 260 kPa. Among the applied techniques, dynamic compaction replacement proved most effective, enhancing the bearing capacity to 580 kPa. However, due to the complex stratigraphy and shallow bedrock in certain zones, dynamic compaction may negatively impact bedrock stability. Numerical modeling further validated that dynamic compaction replacement induced the smallest settlement deformation. Targeted solutions were proposed in response to the issues identified during testing, offering practical guidance for similar foundation treatments in high-fill embankments over expansive soils.

## Introduction

The 14th Five-Year Plan for the Development of Civil Aviation in China emphasizes the strategic expansion and densification of airports in central-western and southern regions, leading to a substantial rise in newly constructed airports. As a result, the challenge of selecting suitable airport sites has gained increasing prominence. To balance technical site specifications with efficient land utilization, a growing number of airports are now being planned and constructed in mountainous terrains. In these areas, extensive earthwork operations—including mountain excavation and valley infilling—are typically necessary to establish level construction platforms^1^. Consequently, high-fill embankment construction has become increasingly common^2–5^. Such projects typically involve considerable fill thicknesses and substantial earthwork quantities, and are frequently associated with insufficient foundation bearing capacity issues^6,7^.

Traditional ground improvement techniques encompass soil replacement, preloading, dynamic compaction, and vibro-compacted gravel columns^8^. A wealth of engineering experience has confirmed the effectiveness of these approaches in markedly improving the bearing capacity of foundations^9–12^. Field assessments of ground improvement effectiveness commonly involve plate load testing, standard penetration tests (SPT), and monitoring of pore water pressure^13–16^. Meng^17^ conducted model experiments to examine the stress transmission behavior of dynamic compaction in both lateral and vertical directions, taking into account parameters such as compaction energy and impact spacing, and subsequently developed a stress attenuation model. Li^18^ performed large-scale outdoor dynamic compaction tests to investigate the impact of various parameters, including energy level, drop height, tamper weight, and tamper diameter, on soil improvement. A statistical correlation between compaction impulse, impact duration, and settlement was developed to inform dynamic compaction construction and quality control practices. Other studies have utilized extensive SPT datasets to formulate advanced settlement prediction models for pile foundations^19^.Wei^20^ conducted field experiments on soil-rock mixed embankment fills treated with dynamic compaction and concluded that dynamic compaction’s effectiveness was limited for embankment fills exceeding 30 m in thickness. Ye and Xu^21^ monitored the ground deformation of saturated silty soil improved by DR. They studied the effect of hammer cushion thickness and particle size of a pillar’s material using different hammer sizes. Tarawneh^22^ believes that when there are non-compacted soil layers in the stratum, granular materials need to be filled into the compaction pits in order to meet the bearing capacity requirements of the foundation. To ensure the long-term stability of compacted geotechnical structures, understanding pore pressure dissipation and determining optimal re-compaction timing are pivotal. Showkat and Babu^23^ explored geocomposite-aided stability under infiltration but left re-compaction timing criteria unresolved. Meanwhile, Showkat and Babu^24^ illuminated anisotropic embankment behavior during infiltration, yet the linkage between pore pressure dissipation dynamics (critical for re-compaction) and safety thresholds remained unclear.

In conclusion, while substantial advancements have been achieved in ground improvement methods, research into the underlying mechanisms—especially those grounded in in-situ experimental data—remains insufficient. This study examines a relocation airport project in Northwest China, focusing on the geotechnical properties of expansive soils in the area. Field tests on dynamic compaction and sand compaction pile were carried out in situ. The experimental results were analyzed to identify the limitations and underlying causes of conventional dynamic compaction, dynamic replacement, and driven gravel piles in treating expansive soils. Numerical simulations were conducted to compare the settlement and deformation behavior of foundations treated with these three techniques. The feasibility and effectiveness of each technique for the project were assessed, and corresponding engineering recommendations and improvement strategies were proposed. The findings offer valuable insights for the treatment of expansive soils in similar high-fill foundation projects.

## Project overview and in-situ test experiments

### Project overview

**Fig. 1:**
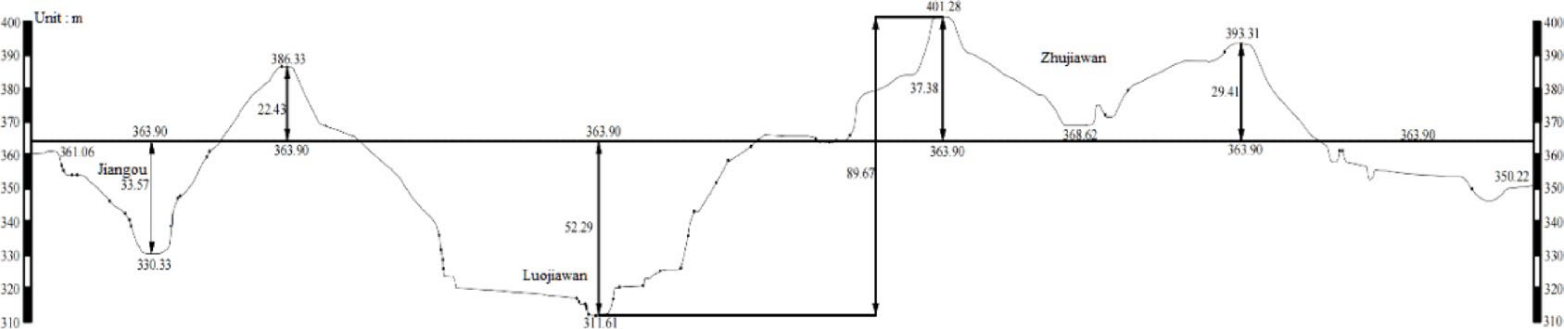
Longitudinal section view of the runway centerline.

The airport runway is 2,600 m in length and 45 m in width. The project site is situated south of the Qinling Mountains, north of the Yue River, and near the Han River. Geomorphologically, the site falls within the terrace-hill transition zone along the northern edge of the Yue River Basin. The area features three ridges and three gullies: Jiangou, Luojiahe, and Zhujiawan. As depicted in the longitudinal profile along the runway centerline, the terrain is highly undulating, with elevations varying between 311 m and 401 m, resulting in a maximum elevation variation of approximately 90 m. The design elevation of the runway centerline embankment is 363.90 m. The site contains two primary fill areas, situated along the north-south flowing Luojiahe and Jiangou gullies. The Luojiahe cross-section features a “U”-shaped valley, with a maximum fill height of approximately 53 m, while the Jiangou cross-section displays a “V”-shaped valley, with a maximum fill height of approximately 33 m. As shown in Fig. 1.

**Fig. 2:**
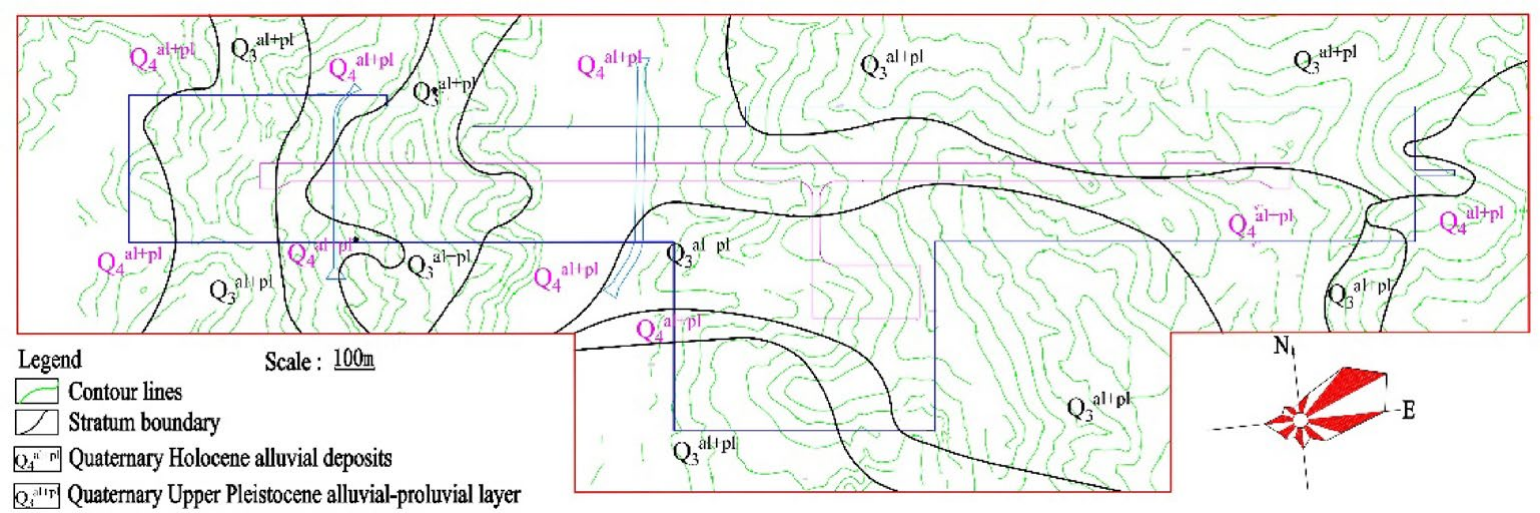
Site area engineering geological map.

The surface strata at the site primarily consist of Quaternary Holocene, Upper Pleistocene, and Middle Pleistocene alluvial and diluvial deposits, including silty clay, cohesive soils, and gravelly soils. These deposits overlie Tertiary strata, including sandy mudstone, sandstone, and conglomerate, underlain by Ordovician phyllite. The engineering geological map of the Ankang Airport site is presented in Fig. 2. The Luojiahe riverbed mainly consists of alluvial clay and cobbles, while sandy mudstone outcrops are found in the Jiangou gully. Expansive soils are extensively distributed in the ridge and terrace areas. The physical and mechanical properties of the primary soil layers at the site are summarized in Table 1.

**Table 1:** Main physical and mechanical parameters of foundation soil.

Soil	γ /kN·m^− 3^	w /%	e	c /kPa	φ /°
Silty clay	18.2 ~ 20.8	17.9 ~ 34.8	0.581 ~ 0.948	35.6	18.8

The project site involves significant areas of cut and fill. To assess the swelling potential of cohesive soils at the site, undisturbed soil samples were collected for laboratory testing. The tests included free swell ratio, swelling ratio under applied pressures of 20 kPa and 50 kPa, shrinkage coefficient, and swelling pressure. The results of the swelling tests are presented in Table 2. Additionally, shrinkage deformation tests were carried out on samples obtained from exploratory boreholes to assess the volumetric shrinkage of the foundation soils. Based on the comprehensive test results and in accordance with the Technical Code for Buildings in Expansive Soil Regions (GB 50112–2013)^25^, the soil in this area is classified as Class I—weakly expansive soil, as indicated in Table 3.

**Table 2:** Expansion test table of foundation soil.

Soil	Statistical items	Maximum	Minimum	Average
Silty clay	*w* /%	34.8	17.4	26.1
	Free expansion rate /%	48.0	24.0	32.75
	Expansion rate(50 kPa)/%	-0.54	-1.19	-0.90
	Contraction coefficient	0.19	0.05	0.10
	Expansion force /kPa	50.0	20.0	30.75

**Table 3:** Shrinkage deformation *S*_*s*_ of foundation soil and scale of expansion and contraction.

Soil	Exploration point	Depth /m	Shrinkage deformation S_s_ /mm	Expansion grade
The foundation of the Luojiahe test section	X12	7.0～10.5	22.00	I
	X14	37.0～40.5	17.20	I
	X15	17.0～20.5	4.85	I
	X44	1.0～4.5	34.73	I
	X47	15.0～18.5	54.20	II
	X50	28.5～32.0	24.23	I
	X53	6.0～9.5	10.15	I
	X56	24.0～27.5	12.30	I
	X81	20.0～23.5	9.85	I
	X87	2.0～5.5	22.45	I
	101	0～3.5	18.90	I

Laboratory and in-situ test results show that the expansive soil at the site has a high natural moisture content, high compressibility, and low expansiveness. Large-scale embankment construction on this type of soil may result in significant differential settlement, potentially impacting both the construction phase and the long-term operation of the airport. Therefore, ground improvement of the native silty clay layer is essential. Trial sections were conducted to compare various foundation treatment schemes. The results of both field and laboratory tests provide a scientific foundation for selecting suitable ground improvement methods for large-scale embankment construction in subsequent phases.

### Test scheme design

Based on site investigation reports, relevant national codes, and extensive engineering experience, three ground improvement methods—conventional dynamic compaction, dynamic replacement, and dynamic compaction combined with driven gravel pile compaction—were chosen for experimental study to treat weak expansive soils with high moisture content and porosity. After ground improvement, the effectiveness of each method was assessed using a combination of laboratory and in-situ tests. Further analysis was performed to explore the underlying reinforcement mechanisms of each technique.

**Fig. 3:**
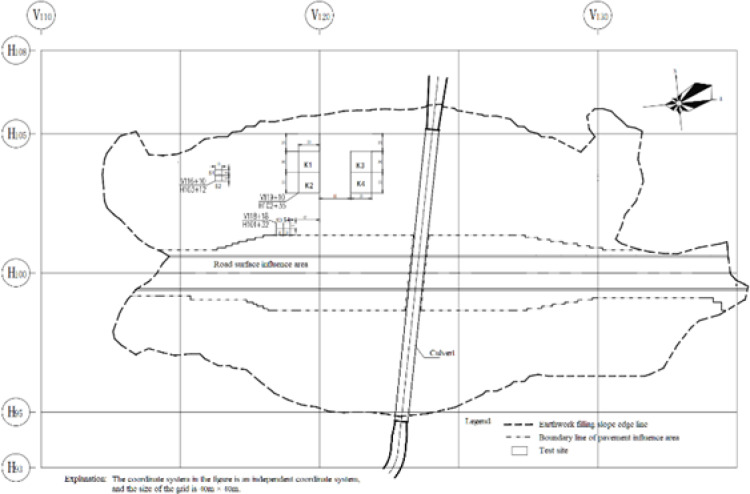
Test area layout plan.

8 test zones were established at the experimental site. 4 zones with high natural moisture content, located in the floodplain area, were selected for driven gravel pile compaction tests, designated as S1, S2, S3, and S4. Additionally, dynamic compaction tests were conducted in 4 zones, including two in the floodplain and two in the hilly terrain, labeled K1, K2, K3, and K4. The layout of the test zones is presented in Fig. 3, and the parameters used in the dynamic compaction tests are shown in Table 4.

**Table 4:** Dynamic compaction test parameters.

Area	Dynamic compaction replacement		Ordinary dynamic compaction	
	K1	K2	K3	K4
Layout form of ramming points /m	5.0 × 5.0	5.0 × 5.0	3.0 × 3.0	3.0 × 3.0
Ramming energy /kN·m	4000	3000	3000	2000
Number of ramming strikes	12 ~ 27	16 ~ 33	9 ~ 18	12 ~ 21
Hammer weight /kg	23,600	23,600	17,600	17,600
Diameter of the hammer /m	1.5	1.5	2.3	2.3
Landing distance /m	17.0	12.7	17.0	11.4

**Fig. 4:**
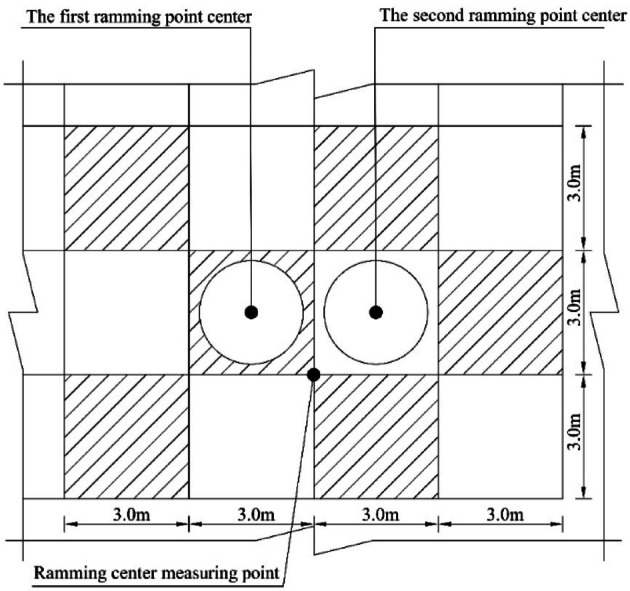
Schematic diagram of interval bump and detection unit.

The dynamic compaction test area underwent a two-pass compaction procedure. The first pass involved jump compaction, followed by backfilling and leveling of the depressions. The second pass was performed using offset jump compaction. The first pass consisted of 16 consecutive blows, and the second pass included 12 consecutive blows. The total number of blows was controlled to ensure that the average settlement of the final two blows did not exceed 5 cm. After point compaction, the site was leveled, and full-area compaction was carried out using an energy level of 1760 kN·m, with a 1/3 overlap between adjacent hammer imprints. The compaction points were arranged in a square grid, with the test area measuring 30 m × 30 m. A schematic diagram of the jump compaction spacing and monitoring units is presented in Fig. 4.

Dynamic compaction replacement involves backfilling the compaction pit upon completion of the initial compaction phase. The backfill material must consist of well-graded block stones and crushed stones to ensure optimal compaction performance. A layer of compacted fill material, with a minimum thickness of 500 mm, should be placed beneath the pier body. The bedding material must match the composition of the pier body material to maintain structural consistency. Subsequently, the pit is backfilled to the level of the pit’s top surface, followed by additional dynamic compaction until the predetermined number of compaction strikes and the required control criteria are satisfied.

**Fig. 5:**
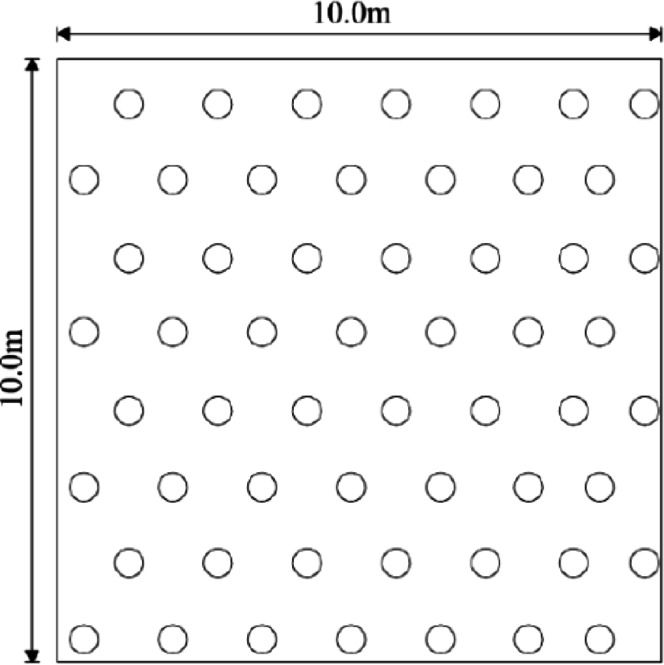
Pile position layout.

The sand compaction pile were arranged in an equilateral triangular pattern, as shown in Fig. 5. The pile material consisted of gravel with particle sizes ranging from 20 to 50 mm, with fines content limited to less than 5%. Upon completion of pile installation, a 0.5 m thick gravel cushion layer was placed on top and compacted with four passes of rolling. The design parameters for the sand compaction pile are listed in Table 5.

**Table 5:** Test parameter of sand compaction pile.

Test area	Pile length /m	Pile diameter /mm	Pile distance /mm
S1	7.0	500	1500
S2	7.0	500	1250
S3	10.0	500	1250
S4	10.0	500	1500

### Experimental test plan

**Fig. 6:**
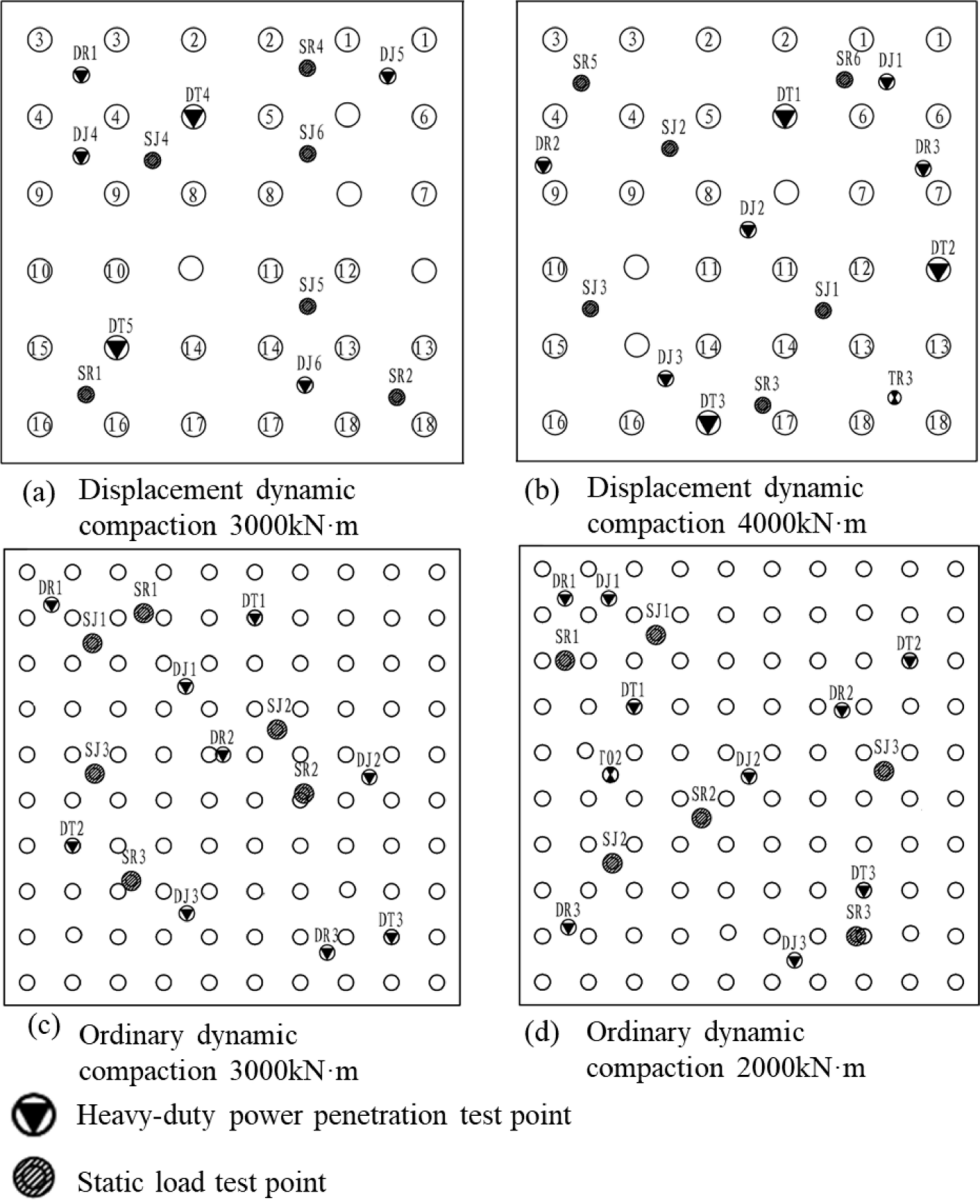
Plan position diagram of dynamic compaction test area.

**Fig. 7:**
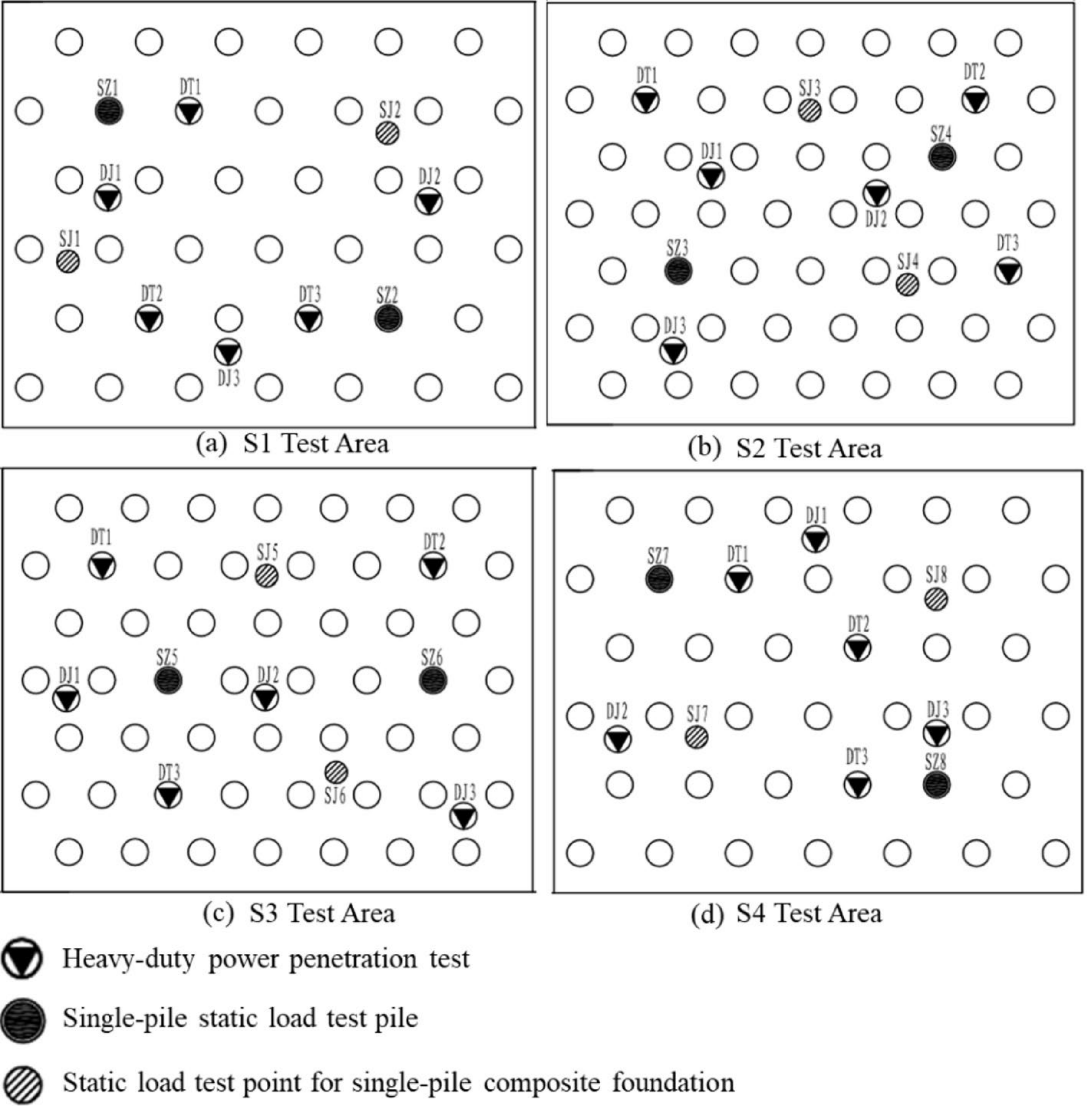
Schematic diagram of the plane position of the test area of sand and gravel pile for submerged tube compaction.

Field sampling and corresponding laboratory geotechnical tests were conducted before and after the implementation of dynamic compaction and driven gravel pile compaction. Additionally, in-situ tests including shallow plate load tests, standard heavy dynamic penetration tests, and pore water pressure measurements were performed. The effectiveness of dynamic compaction and sand compaction pile in reducing high moisture content and expansive behavior was evaluated by comparing changes in the physical and mechanical properties, strength, and deformation parameters of the expansive soil layer before and after treatment. The layout of the test measurement points is presented in Figs. 6 and 7.

Testing of physical and mechanical properties:

1. Boreholes were drilled in the test zones before and after dynamic compaction to collect samples. In each test zone, three boreholes were located at the center of each compaction point. Post-compaction, soil samples were collected at 1.0 m intervals from the surface for standard geotechnical testing. For comparison with the natural (fill) state, three additional boreholes were drilled in each test zone prior to compaction. Soil samples were collected every 1.0 m from the natural ground surface for both standard geotechnical and swelling tests.
2. Shallow plate load testing: Shallow plate load tests were performed 7 to 14 days after the completion of dynamic compaction. These tests were conducted to evaluate the bearing capacity and deformation characteristics of the foundation. A summary of the plate load test results is provided in Table 6.
3. Standard penetration testing (SPT) using heavy dynamic penetration: In each test zone, three heavy dynamic penetration tests were conducted on the natural soil. After compaction, three tests were conducted at the center of each main compaction point and three between adjacent points, including four tests at central positions.
4. Pore water pressure measurement: Pore water pressure was monitored at three locations in the K1 test zone. Dynamic compaction was applied in two passes. Before the second compaction pass, sufficient pore pressure dissipation was required; otherwise, further compaction could trigger “mud pumping” or “soft slurry” conditions, preventing the soil from achieving the intended reinforcement effect. Pore pressure monitoring was used to determine the optimal interval between compaction passes.

**Table 6:** Overview of plate load test.

Area			D /m	Energy /kN	Loading quantity /kPa	
Sand compaction pile	S1		1.58	2000	70	
	S2		1.31	2000	70	
	S3		1.31	2000	70	
	S4		1.58	2000	70	
Dynamic compaction	K1	Natural	0.8	100	80	
		Compacted	1.58	3200	Point 100	Interval 60
	K2	Natural	0.8	100	80	
		Compacted	1.25	3200	Point 100	Interval 60
	K3	Natural	1.13	500	125	
		Compacted	1.58	3200	70	
	K4	Natural	1.13	1000	40	
		Compacted	1.58	3200	80	

## Analysis and evaluation of test results

Soil samples were obtained from three test zones both before and after ground improvement. Laboratory tests on physical and mechanical properties, along with in-situ testing, were performed. The resulting data supported foundation settlement calculations and were used to assess the performance of various ground improvement methods.

### Comparative analysis of physical and mechanical property indicators of soil mass

A comparison was conducted between the mechanical properties of foundation soils improved by dynamic compaction and sand compaction pile and those of untreated natural soils. Table 7 summarizes the physical and mechanical properties of foundation soils treated with conventional dynamic compaction and dynamic replacement.

**Table 7:** Statistical of physical and mechanical property indexes of foundation soil before and after treatment.

Area	Ordinary dynamic compaction		Dynamic compaction replacement		Sand compaction pile	
Type	Natural	Compacted	Natural	Compacted	Natural	Compacted
*w*_*max*_/%	17.0	15.0	17.0	16.0	17.0	15.7
*ρ*_*d, max*_/g/cm^3^	1.66	1.79	1.66	1.71	1.67	1.75
*e*	0.774	0.869	0.794	0.630	0.781	0.623
*S*_*r*_/%	97.3	87.0	92.0	76.0	85.0	87.7
*E*_*s*_/MPa	5.21	13.75	5.37	9.82	5.27	11.1
*c*/kPa	30.8	81.4	32.7	59.4	31.2	71.7
*φ*/°	14.4	18.4	15.6	24.7	15.1	20.3

As indicated in Table 7, the optimum moisture content of reinforced foundation soils is lower than that of natural soils, while the maximum dry density is correspondingly higher. This can be attributed to the compaction energy introduced by the three improvement techniques, which facilitates the rearrangement of soil particles in expansive soils and promotes the aggregation of fine particles.

The dynamic compaction replacement method achieves a higher maximum dry density compared to both sand compaction pile and conventional dynamic compaction, and exhibits the lowest optimum moisture content. This is because both dynamic replacement and driven gravel pile methods partially replace expansive soils, and the high compaction energy during construction crushes part of the replacement material into finer particles. These finer particles then aggregate with the surrounding expansive soils. In contrast, conventional dynamic compaction compresses the expansive soil solely through impact energy, resulting in a lower dry density compared to the other two methods.

In engineering practice, although the exact optimum moisture content and maximum dry density are rarely achieved, the moisture content can be artificially controlled to bring the soil closer to its maximum dry density.

A comparative analysis of the compression modulus of foundation soils treated by conventional dynamic compaction, dynamic replacement, and sand compaction pile, as well as untreated natural soils, revealed the following results: the compression modulus increased by 83% after conventional dynamic compaction, by 164% following dynamic replacement, and by 111% for the pile-soil system treated with sand compaction pile, all relative to the untreated soil.

These results indicate that dynamic replacement provides a more pronounced improvement in foundation stiffness than the other two methods. Regarding shear strength parameters, the cohesion of the treated soil increases most notably, whereas the internal friction angle shows only a modest increase, with dynamic replacement exhibiting the most substantial enhancement among the three techniques.

### In-situ test results

#### Shallow plate load test

**Fig. 8:**
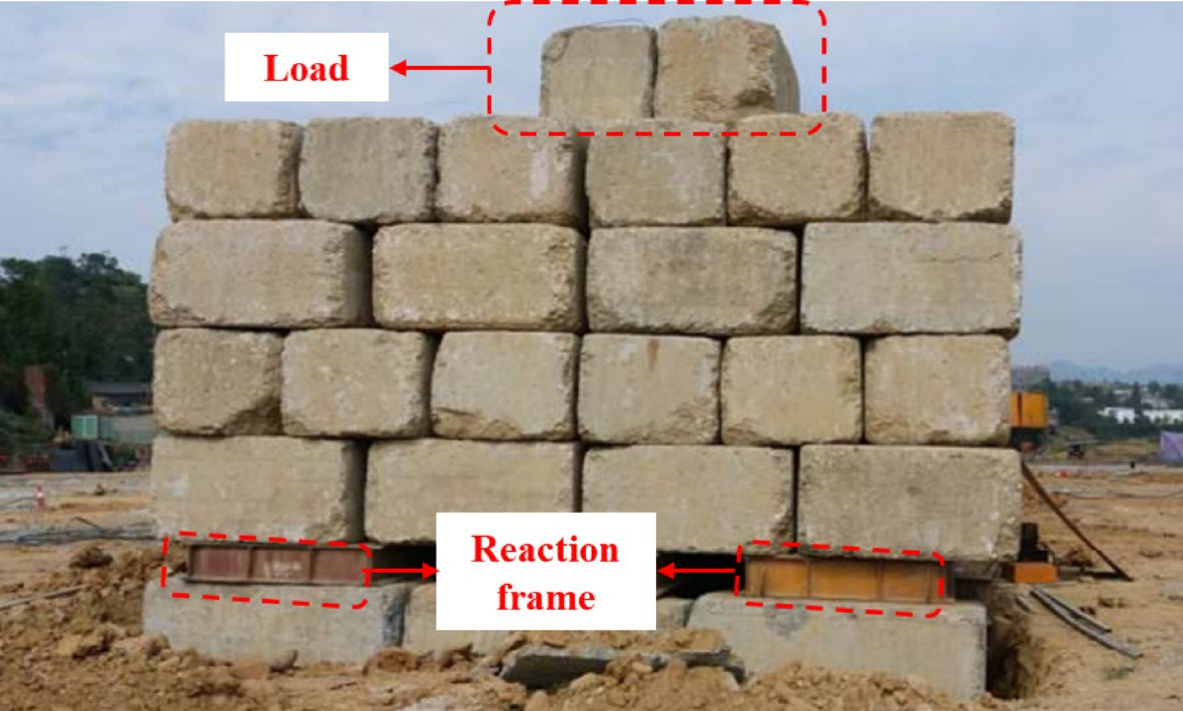
Site static load test diagram.

**Fig. 9:**
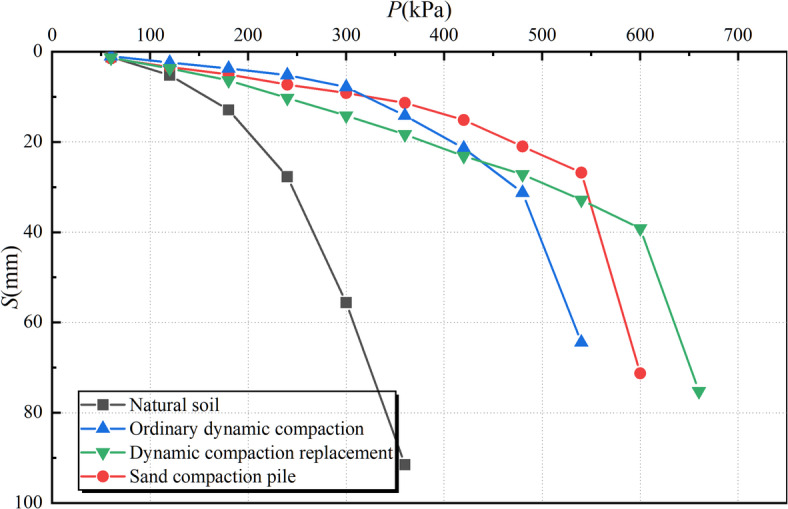
Comparison of P-S curves for static load test.

The project involved eight test zones, where static load tests were carried out on composite foundations reinforced with sand compaction pile, on individual sand compaction pile, and on dynamic compaction impact points. A circular steel bearing plate was used in all tests, with detailed test parameters listed in Table 6. Figure 8 illustrates the site layout for static load testing, and Fig. 9 compares the *P–S* curves for the three foundation improvement methods and untreated soil.

As illustrated in Fig. 9, the characteristic bearing capacity of the untreated soil is 260 kPa. The foundation treated with conventional dynamic compaction achieves 450 kPa, dynamic replacement reaches 580 kPa, and the driven gravel pile foundation achieves 640 kPa. All three ground improvement methods significantly enhanced the bearing capacity of the foundation, with detailed improvement values summarized in Table 8.

**Table 8:** Bearing capacity characteristic value results.

Value	Characteristic value of bearing capacity /kPa	Increase amplitude /%
Natural foundation	260	-
Ordinary dynamic compaction	450	73.1
Dynamic compaction replacement	580	123.1
Sand compaction pile	640	146.2

#### Heavy-duty dynamic probing test

A comparison of the dynamic probing N_63.5_ values at the center of the impact points and the pre-compaction soil, along with the improvement magnitude, is presented in Table 9.

**Table 9:** Comparison of the number of dynamic penetration N_63.5_ before and after tamping.

Experimental area		Depth/m	Natural foundation	Tamping point		Main compaction point	
			Average	Average	Increase amplitude /%	Average	Increase amplitude /%
Dynamic compaction replacement	K1	Silt	2.6	3.2	23.1	37.3	/
		Gravel sand	6.2	11.2	80.6		
		Mudstone	35.5	26.8	-24.5		
	K2	Silt	2.6	3.7	42.3	36.9	/
		Gravel sand	6.2	10.9	75.8		
		Mudstone	35.5	26.9	-24.2		
Ordinary dynamic compaction	K3	0.0 ~ 1.2	15.9	7.4	-53.5	9.2	-42.1
		1.3 ~ 2.1	37.9	14.8	-60.9	18.0	-52.5
		2.2 ~ 2.7	/	27.5	/	27.1	/
	K4	0.0 ~ 0.8	8.0	12.1	51.3	14.4	80.0
		0.9 ~ 2.5	14.0	22.9	63.6	26.7	90.7
		2.6 ~ 6.5	33.0	32.7	-0.9	37.1	12.4

n the K1 and K2 zones, the compaction of the pile-soil between impact points resulted in a slight increase in soil density following dynamic replacement compaction. The blow count in the silt layer increased by approximately 32.7%, while in the mudstone or gravel-sand layers, it increased by approximately 78.2%. In the K3 zone, dynamic compaction affected the shallow mudstone layer, resulting in bedrock fracturing. Consequently, the dynamic probing blow count in the pile-soil and impact points was lower than that of the untreated soil. In the K4 zone, the effective depth of dynamic compaction ranged from 3.0 to 4.0 m. At this depth, the dynamic probing blow count for the untreated soil overlapped with that of the pile-soil and impact points, indicating significant reinforcement effects. The blow count increased by more than 80% above 2.5 m, while the improvement below 2.5 m remained limited.

**Table 10:** Comparison of the number of dynamic explorations and hits in the test area of sand compaction pile.

Experimental area	Depth/m	Natural foundation	Inter-pile		Pile body	
		Average	Average	Increase amplitude /%	Average	Increase amplitude /%
S1	0.0 ~ 1.9	2.8	4.1	46	12.4	343
	2.0 ~ 5.0	5.4	6.2	15	8.8	63
	5.1 ~ 7.0	22.7	26.2	15	27.8	22
S2	0.0 ~ 1.9	2.8	7.1	154	13.0	364
	2.0 ~ 4.0	5.4	7.0	30	10.6	96
	4.1 ~ 5.6	22.7	19.6	-14	24.3	7
S3	0.0 ~ 1.9	2.8	4.6	64	10.9	289
	2.0 ~ 4.2	5.4	6.9	28	10.2	89
	4.3 ~ 5.9	22.7	23.7	4	25.5	12
S4	0.0 ~ 3.9	2.8	4.9	75	9.6	243
	4.0 ~ 5.2	5.4	13.5	150	16.7	209
	5.3 ~ 6.0	22.7	29.8	31	35	54

As indicated in Table 10, the vibratory pipe-jacked gravel pile exhibited a significant reinforcement effect on shallow soils. The blow count within the pile increased by approximately 309.8%, while the blow count in the surrounding pile-soil rose by about 84.8%. However, due to the already compact nature of the deeper strata, reinforcement at depth was relatively limited. The blow count in the deeper portion of the pile increased by around 114.2%, and by approximately 55.8% in the adjacent soil. In the S2 zone, a negative change in blow count at the pile base was observed, likely due to damage to the competent bedrock layer caused during pile installation.

The increase in the number of dynamic touch strikes in the K2, K3 and S2 regions is negative. In this area, there are shallow bedrock layers. The compaction vibration causes the rock mass fractures to expand until they are destroyed. High-energy dynamic compaction leads to the destruction of the soil framework and the loss of strength. All these will lead to insufficient bearing capacity of the foundation, causing uneven settlement and deformation. During the construction of the project, supplementary investigation is required. Geological radar or core drilling sampling should be adopted to confirm whether the bedrock has been damaged. Other measures such as grouting should be adopted to reinforce fractured rock masses. Long-term monitoring points should also be set up.

**Fig. 10:**
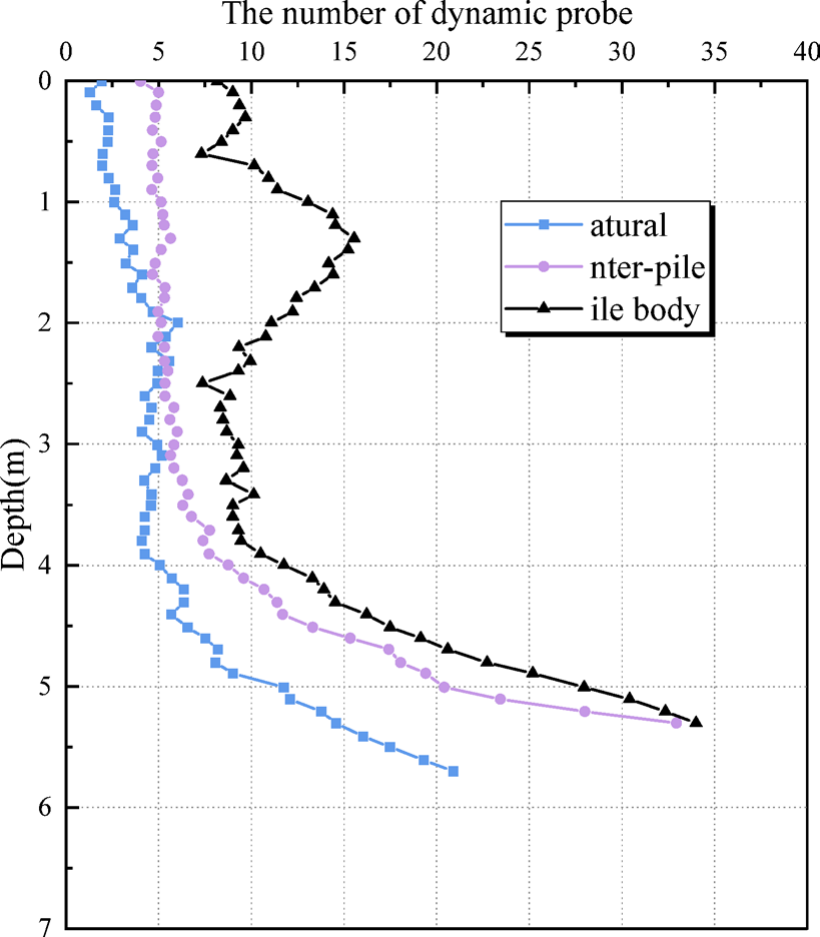
Comparison of the number of strikes between piles, piles and natural soil.

**Fig. 11:**
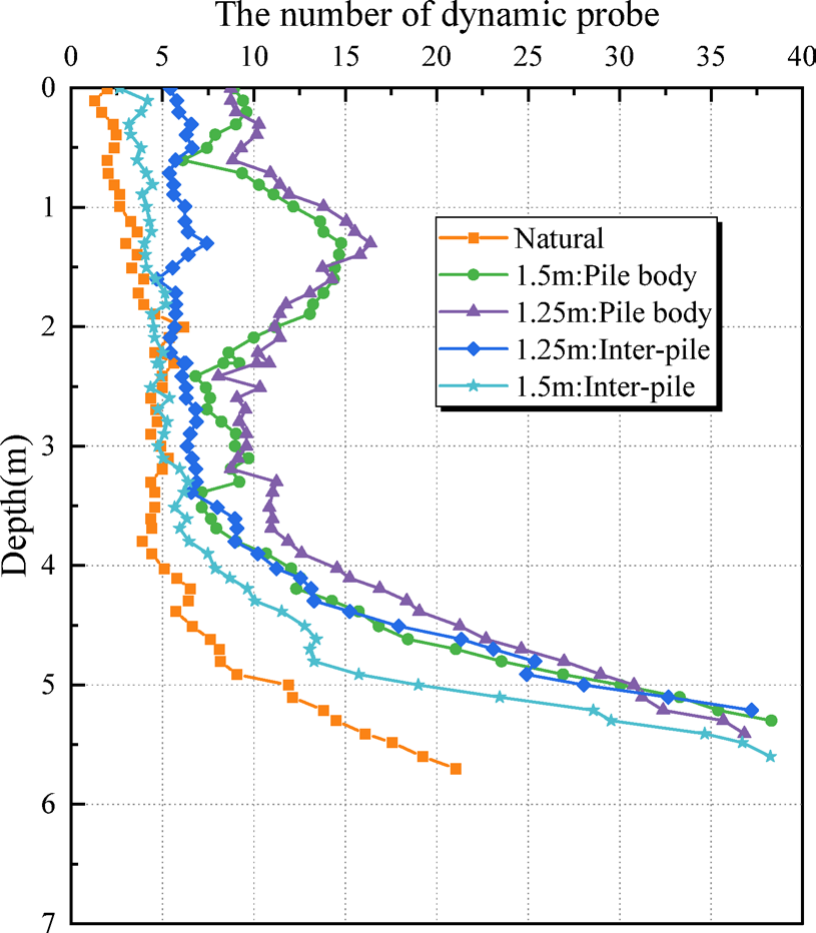
Comparison of stroke numbers of different pile distances.

As illustrated in Fig. 10, the increase in blow count within the pile is generally higher than that observed in the surrounding soil, and this difference is particularly pronounced in the shallow layers. Figure 11 demonstrates a positive correlation between pile spacing and blow count in the shallow foundation soils.

#### Pore water pressure test

**Fig. 12:**
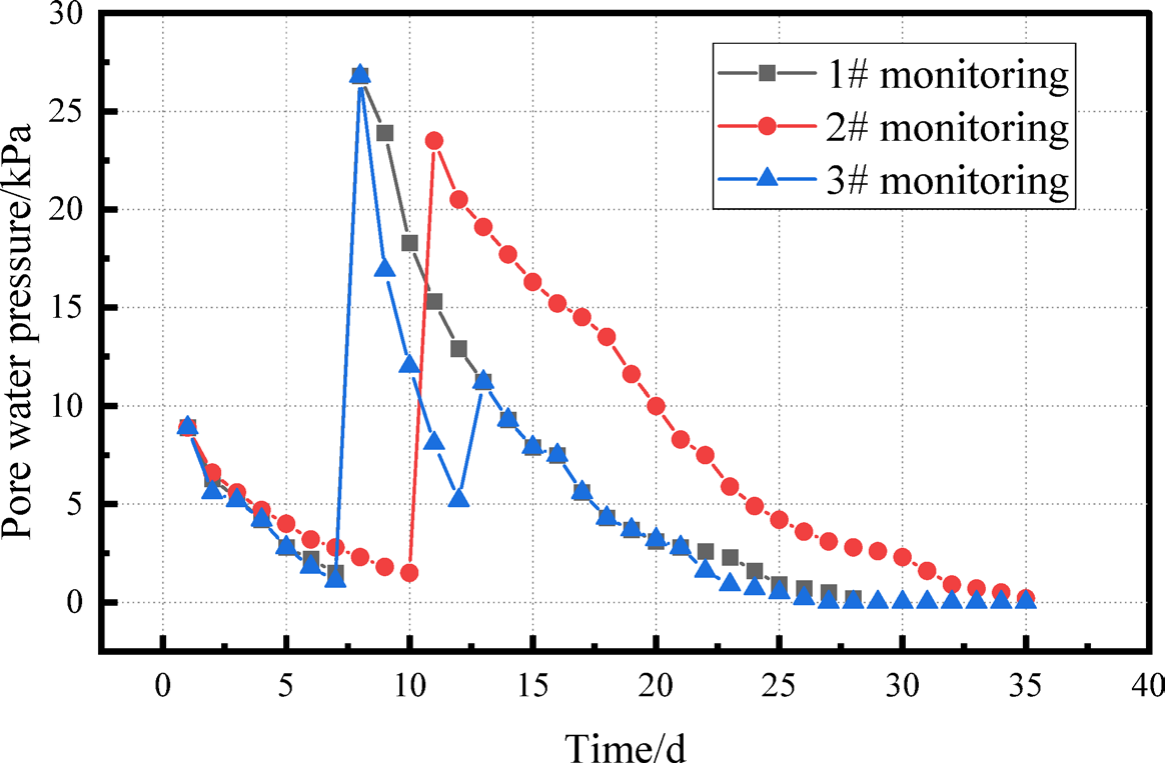
Pore water pressure dissipation diagram.

The trend of pore water pressure variation, derived by subtracting initial values from the measured data, is presented in Fig. 12 as a function of observation time. It is evident that pore water pressure rises sharply immediately after each dynamic compaction event and then gradually dissipates with time. In test zones K1 and K2, more than 80% of the excess pore water pressure dissipated within 7–15 days following the first round of dynamic compaction, and within 5–12 days after the second round. By 25–30 days, approximately 95% of the excess pore pressure had dissipated.

Secondary compaction should be carried out when the pore water pressure has fully dissipated and the effective stress of the soil has stabilized. If it is done too early, the secondary loading will superimpose the pore water pressure, causing the soil to deform out of control. If it is too late, the rebound of soil or the redistribution of moisture will reduce the compaction efficiency and even lead to “false compaction”.

### Analysis of reinforcement mechanism

**Fig. 13:**
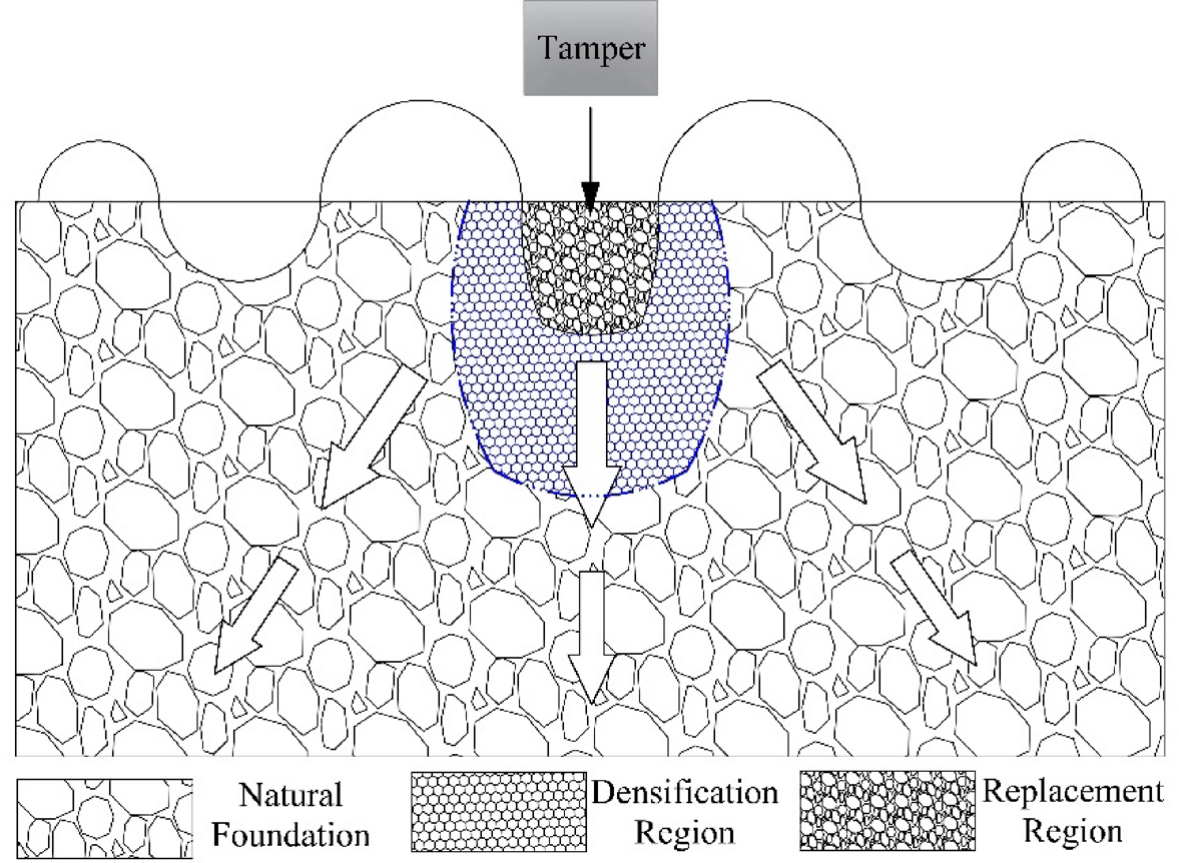
The load transfer mechanisms of the conventional dynamic compaction method and the dynamic compaction displacement method.

**Fig. 14:**
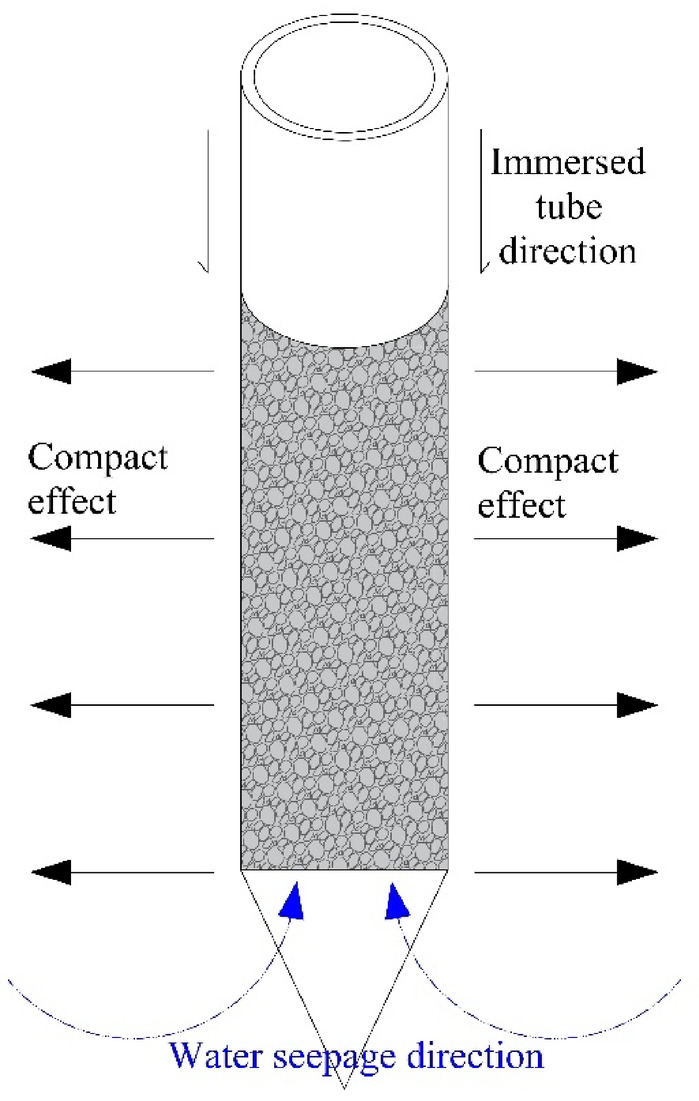
The load transfer mechanism of sand compaction pile.

(1) Ordinary dynamic compaction reinforces the foundation through dynamic compaction. Conventional dynamic compaction converts the potential energy of the rammer into kinetic energy, which is then transferred to the foundation soil to form transverse and longitudinal waves. Transverse waves cause shear failure but do not lead to volume deformation. Longitudinal waves cause volume changes in the soil along the direction of wave propagation. The core of it is to promote the directional migration and rearrangement of soil particles through impact energy. The shock wave forcibly compresses the pores between soil particles, transforming the particles from a loose and disordered state into a compact framework structure. After dynamic compaction, the soil undergoes disturbance and reaches a new equilibrium state, with an increase in thixotropic strength^26^. Therefore, the combined effect of failure and static compaction strengthens the soil. Due to the limited reinforcement range of the rammer, reinforcement zones and elastic zones will be formed within its influence range. The load transfer mechanism of the common dynamic compaction method is shown in Fig. 13.

(2) Dynamic compaction displacement method forms a composite foundation through dynamic displacement. The mechanism is to use a heavy hammer to impact the weak foundation soil, forming a compaction pit. In the compaction pit, coarse-grained materials such as sand, gravel and crushed stones are filled to form a pier body. The pier body and the soil between the piers bear the load through stress sharing and coordination. The pore water in the foundation soil is discharged through the pier body, promoting the consolidation of the foundation soil and thereby enhancing its bearing capacity. The load transfer mechanism of the dynamic compaction displacement method is shown in Fig. 13.

(3) During the construction process of the immersed tube compacted sand and gravel pile, the pile tube is pressed into the soil layer through vibration, squeezing the surrounding soil, reducing the pore ratio of the loose sand and soil, and increasing its density and shear strength. The pile body itself has replaced part of the soft soil, forming a high-strength pile body that shares the load with the surrounding soil. The excellent permeability of sand and gravel piles also accelerates the drainage of pore water in the foundation soil, promotes soil consolidation, and enhances long-term strength. The pile structure also has a reinforcing effect, forming a rigid reinforcing body in the soil, restricting the lateral deformation of the soil, and improving the stability of the foundation. The load transfer mechanism of the immersed tube compacted sand and gravel pile is shown in Fig. 14.

## Foundation settlement calculation based on field test parameters

Foundation settlement behavior was evaluated through finite element analysis, and the performance of three ground improvement methods was comparatively assessed based on the resulting settlement profiles. These findings offer both theoretical guidance and practical insights for on-site implementation and future applications in similar geotechnical engineering projects.

### Model establishment and boundary conditions

**Fig. 15:**
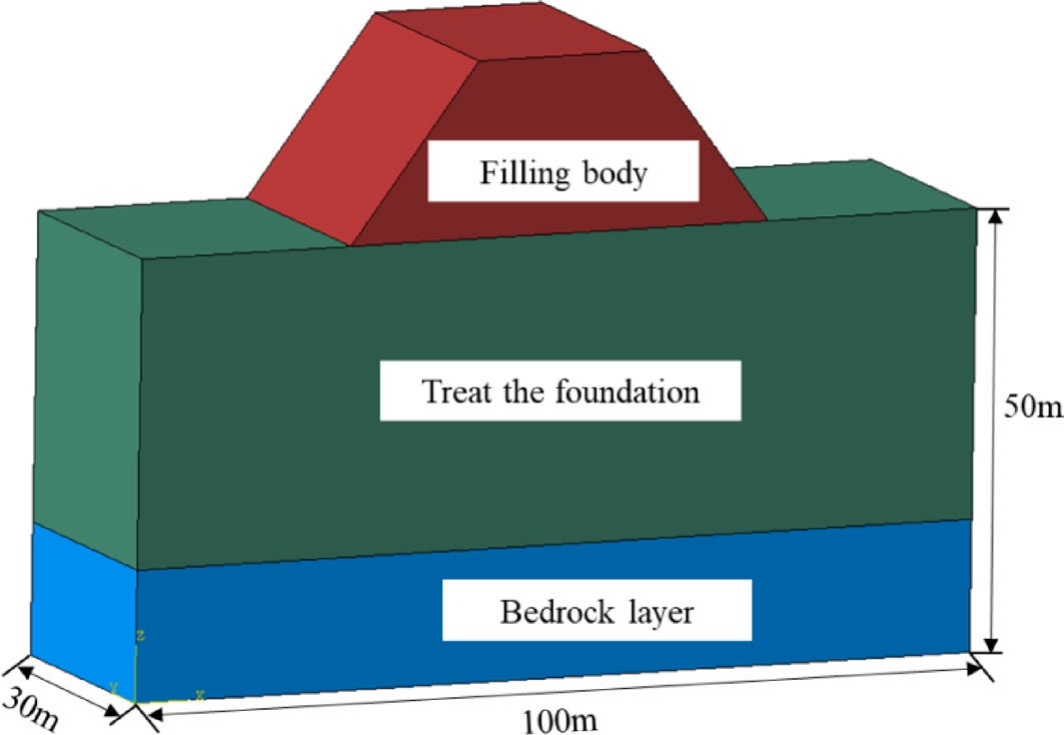
Foundation calculation model.

Considering the actual site conditions, a comparative evaluation was performed on the settlement responses of three ground improvement techniques and untreated natural soil. The finite element model used for the analysis is illustrated in Fig. 15, detailing the material stratification and dimensions of the computational domain.

Numerical simulations were carried out using ABAQUS finite element software. An idealized elastoplastic constitutive model was adopted, with material parameters summarized in Table 11, and the Mohr–Coulomb failure criterion was used to simulate soil behavior. Full displacement constraints were applied at the model base, while lateral boundaries were constrained in the normal direction. The mesh consisted of 19,500 C3D8 (eight-node linear brick) elements.

**Table 11:** Soil property calculation parameter.

Soil type	E/MPa	γ /kN/m^3^	ν	c/kPa	φ /°
Natural foundation	15.6	19.9	0.33	31.6	15.0
Ordinary dynamic compaction	30.2	20.6	0.32	59.4	24.7
Dynamic compaction replacement	42.3	21.3	0.30	81.4	18.4
Sand compaction pile	35.0	21.1	0.30	71.7	20.3
Filling body	26.0	19.5	0.32	48.5	24.7
Bedrock layer	3 × 10^5^	25.0	0.20	-	-

### Analysis of foundation settlement deformation

**Fig. 16:**
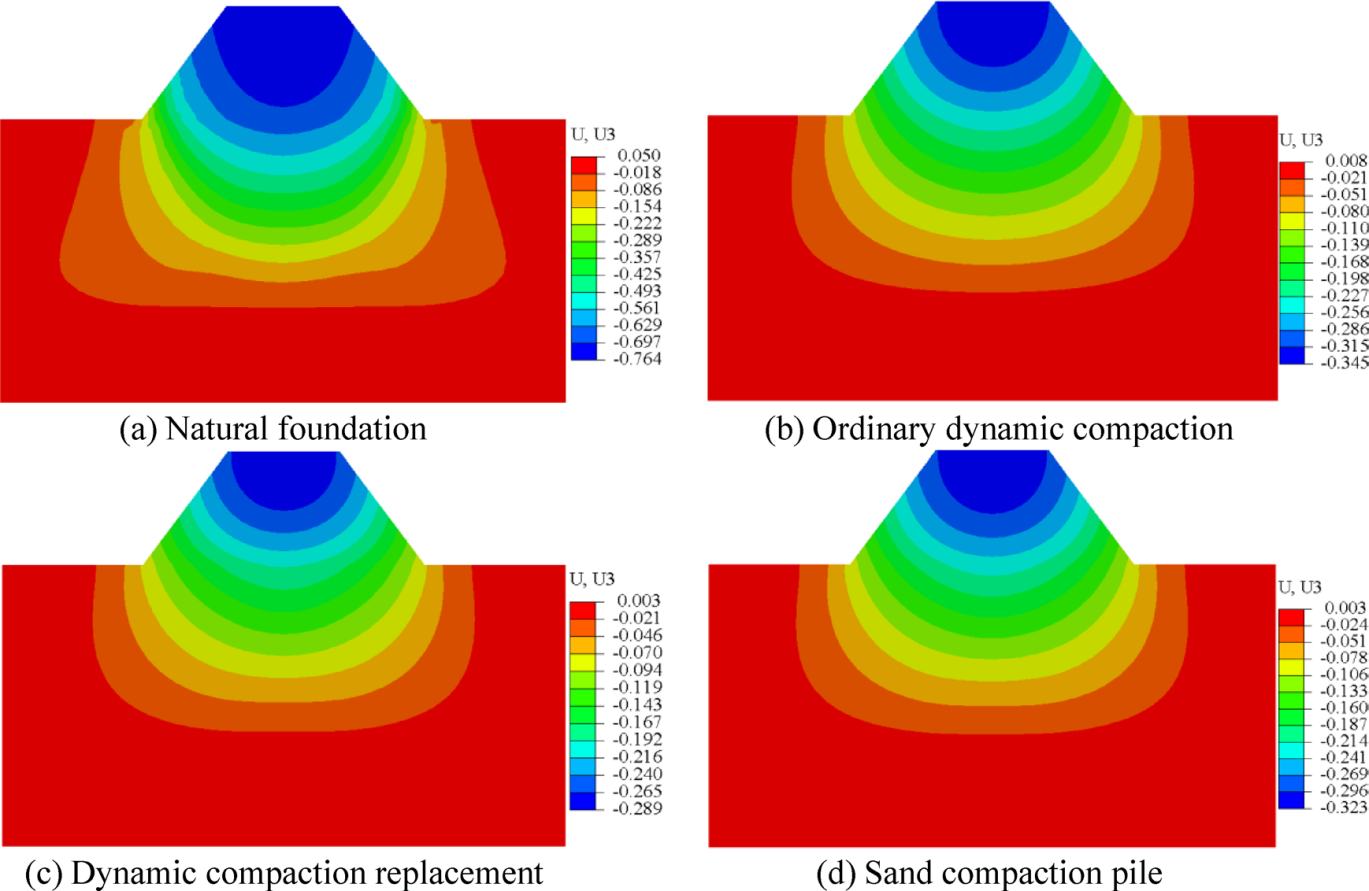
Settlement cloud map after foundation treatment.

Settlement contour plots corresponding to the three foundation improvement methods, obtained from finite element simulations, are presented in Fig. 16.

Figure 16 presents the settlement deformation contour plots for both the natural soil and the three foundation treatment methods. It is evident that, under the load of the high embankment fill, the maximum settlement deformation occurs at the center of the foundation. The natural soil foundation experiences a maximum settlement of 76.4 mm, whereas the foundation treated with dynamic compaction replacement shows a maximum settlement of 28.9 mm. The detailed calculation results are summarized in Table 12.

**Table 12:** Statistical of foundation settlement.

Foundation treatment plan	Settlement volume /mm
Natural foundation	76.4
Ordinary dynamic compaction	34.5
Dynamic compaction replacement	28.9
Sand compaction pile	32.3

As shown in Fig. 16; Table 12, the dynamic compaction replacement method proves to be the most effective reinforcement technique among the three foundation treatment approaches. This method utilizes high-impact energy to disrupt the cementation points and contact surfaces between soil particles, allowing the particles to rearrange into a denser structure. Additionally, the backfilling of coarse sand and gravel into the compaction pits creates additional drainage channels, facilitating the rapid expulsion of pore water and accelerating the consolidation process. Overall, the dynamic compaction replacement method is the most suitable solution for foundation treatment in this project.

## Conclusion

This study addresses the foundation treatment challenges of high embankments in relocation projects by investigating the engineering properties of expansive soils in the region through surveys and tests. Additionally, research was conducted on the use of dynamic compaction for soil improvement. The following conclusions were drawn, along with suggestions for future large-scale construction:

1. The clay at the site exhibits high moisture content and weak expansiveness, with a natural characteristic bearing capacity of 260 kPa. Foundation treatment beneath high embankments is crucial.
2. Both field and laboratory tests confirmed that dynamic compaction replacement is the most effective method for improving the foundation soil in this region, as it improves the foundation’s bearing capacity and mitigates the expansive soil’s impact on the embankment. This method outperforms both ordinary dynamic compaction and vibratory pipe-jacking stone gravel piles. The specific parameters of the dynamic compaction displacement method are shown in Table 4.
3. Numerical simulations based on field and laboratory test results indicated that, after high embankment construction, the maximum settlement of the foundation occurs at its center. Settlement deformation is minimized when using dynamic compaction replacement.
4. Field test results indicate that the foundation layers in the area are complex. If the high-energy dynamic compaction replacement method were fully applied, the foundation’s bearing capacity in some areas could be negatively affected. In some areas, the soil layer above the bedrock is thin, and using pile body replacement may destabilize the bedrock. Therefore, based on geological investigation data, this project ultimately selected a combination of ordinary dynamic compaction and dynamic compaction replacement for foundation treatment.

## Data Availability

For the data of the results of this study, please contact Hou Bin. 13359214651@163.com.
